# Dysregulation of Lipid Metabolism in Mkp-1 Deficient Mice during Gram-Negative Sepsis

**DOI:** 10.3390/ijms19123904

**Published:** 2018-12-06

**Authors:** Jinhui Li, Xiantao Wang, William E. Ackerman, Abel J. Batty, Sean G. Kirk, William M. White, Xianxi Wang, Dimitrios Anastasakis, Lobelia Samavati, Irina Buhimschi, Leif D. Nelin, Markus Hafner, Yusen Liu

**Affiliations:** 1 Center for Perinatal Research, The Research Institute at Nationwide Children’s Hospital, Columbus, OH 43215, USA; jinhui.liu@nationwidechildrens.org (J.L.); Abel.Batty@nationwidechildrens.org (A.J.B.); Sean.Kirk@nationwidechildrens.org (S.G.K.); white.2729@buckeyemail.osu.edu (W.M.W.); xianxi.wang@nationwidechildrens.org (X.W.); Irina.Buhimschi@nationwidechildrens.org (I.B.); leif.nelin@nationwidechildrens.org (L.D.N.); 2 Laboratory of Muscle Stem Cells and Gene Regulation, National Institute of Arthritis and Musculoskeletal and Skin Disease, National Institutes of Health, Bethesda, MD 20892, USA; xiantao.wang@nih.gov (X.W.); dimitrios.anastasakis@nih.gov (D.A.); markus.hafner@nih.gov (M.H.); 3 Department of Obstetrics and Gynecology, The Ohio State University College of Medicine, Columbus, OH 43210, USA; William.Ackerman@nationwidechildrens.org; 4 Center for Molecular Medicine and Genetics, Wayne State University School of Medicine, Detroit, MI 48201, USA; lsamavat@med.wayne.edu; 5 Department of Pediatrics, The Ohio State University College of Medicine, Columbus, Ohio 43205, USA

**Keywords:** *E. coli* infection, sepsis, liver steatosis, hypertriglyceridemia, Mkp-1

## Abstract

Mitogen-activated protein kinase phosphatase (Mkp)-1 exerts its anti-inflammatory activities during Gram-negative sepsis by deactivating p38 and c-Jun N-terminal kinase (JNK). We have previously shown that *Mkp-1*^+/+^ mice, but not *Mkp-1*^−/−^ mice, exhibit hypertriglyceridemia during severe sepsis. However, the regulation of hepatic lipid stores and the underlying mechanism of lipid dysregulation during sepsis remains an enigma. To understand the molecular mechanism underlying the sepsis-associated metabolic changes and the role of Mkp-1 in the process, we infected *Mkp-1*^+/+^ and *Mkp-1*^−/−^ mice with *Escherichia coli* i.v., and assessed the effects of *Mkp-1* deficiency on tissue lipid contents. We also examined the global gene expression profile in the livers via RNA-seq. We found that in the absence of *E. coli* infection, *Mkp-1* deficiency decreased liver triglyceride levels. Upon *E. coli* infection, *Mkp-1*^+/+^ mice, but not *Mkp-1*^−/−^ mice, developed hepatocyte ballooning and increased lipid deposition in the livers. *E. coli* infection caused profound changes in the gene expression profile of a large number of proteins that regulate lipid metabolism in wildtype mice, while these changes were substantially disrupted in *Mkp-1*^−/−^ mice. Interestingly, in *Mkp-1*^+/+^ mice *E. coli* infection resulted in downregulation of genes that facilitate fatty acid synthesis but upregulation of Cd36 and Dgat2, whose protein products mediate fatty acid uptake and triglyceride synthesis, respectively. Taken together, our studies indicate that sepsis leads to a substantial change in triglyceride metabolic gene expression programs and Mkp-1 plays an important role in this process.

## 1. Introduction

Severe sepsis and septic shock are a major cause of death in the United States, accounting for 215,000 deaths and 750,000 hospitalizations annually [1]. The mortality rate of septic shock still approaches 50% despite improvements in critical care medicine [2]. Metabolic dysregulation has been reported in septic patients [3,4,5,6] as well as in experimental animals such as sheep [7], dogs [8], and rodents following sepsis induction [9,10]. Septic patients or animals with Gram-negative bacteria infection often develop hyperlipidemia [11,12,13], which is often referred to as the lipemia of sepsis [14]. Since triglyceride-rich lipoproteins can bind and sequester endotoxin, hyperlipidemia of sepsis is considered a component of the innate, non-adaptive host immune response to infection [14]. In addition to the ability to sequester lipopolysaccharide (LPS), certain fatty acid species interfere with the inflammatory signaling pathway mediated by nuclear factor (NF)-κB [15]. However, derivatives of fatty acids such as arachidonic acid and prostaglandins are important mediators of the systemic inflammatory response, raising the possibility that hyperlipidemia may also be contributing factor to the pathophysiology of sepsis [16]. This is supported by a small study that found a higher incidence of hypertriglyceridemia among non-survivors than among survivors [6]. As septic patients and animals have decreased lipoprotein lipase activity in peripheral tissues such as heart, muscle, and adipose tissue as the result of elevated tumor necrosis factor (TNF)-α levels in the circulation, hypertriglyceridemia during sepsis has been attributed to defective triglyceride clearance in peripheral tissues [17,18,19,20]. However, increased very low density lipoprotein (VLDL)-mediated triglyceride efflux from the liver may also contribute to sepsis-mediated hypertriglyceridemia [21]. In fact, hepatocytes isolated from mice challenged with endotoxin exhibit increased secretion of VLDL, likely as a result of increased expression of apolipoprotein (Apo) b48, Apob100, and microsomal triglyceride transfer proteins [22], the proteins critical to VLDL assembly. One study has shown that TNF-α challenge in mice elevates the hepatic expression of sterol regulatory element-binding transcription factor (Srebf) 1 (also referred to as SREBP-1), a master regulator of lipogenesis and fatty acid synthase (Fasn), which controls the synthesis of saturated fatty acids [23]. However, another study has reported a decreased expression of lipogenesis genes in endotoxin-challenged mice [24]. The mechanisms of hepatic lipid regulation during sepsis remain poorly understood.

Mkp-1 exerts its anti-inflammatory effects by dephosphorylating p38 and JNK during gram-negative bacteria infection [25,26,27,28,29,30]. Previously, we have found that hypertriglyceridemia occurs in *E. coli*-infected *Mkp-1*^+/+^ mice, but not in *Mkp-1*^−/−^ mice [10], suggesting that Mkp-1 plays an essential role in the sepsis-induced hypertriglyceridemia. To understand the molecular mechanisms by which Mkp-1 regulates lipid metabolism during sepsis, we assessed the liver lipid contents and the global gene expression profiles in wildtype and Mkp-1 deficient mice before and after sepsis induction. We found that *E. coli* infection enhanced liver triglyceride synthesis in an Mkp-1-dependent manner with an attenuation of the transcriptional program responsible for fatty acid synthesis and fatty acid oxidation. Our studies also suggest that sepsis likely exacerbates liver lipid accumulation through both increased liver fatty acid uptake and decreased fatty acid β-oxidation.

## 2. Results

### 2.1. Changes in p38 Activity and Dysregulation of Lipid Metabolism Caused by Mkp-1 Deficiency and E. coli Infection

**Figure 1:**
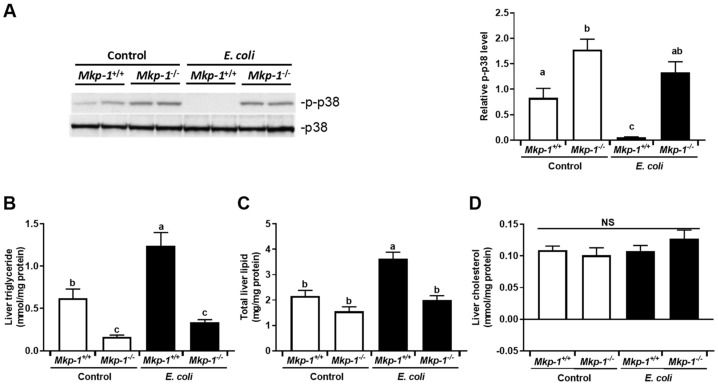
Liver p38 activity and lipid contents before and after *E. coli* infection. *Mkp-1*^+/+^ and *Mkp-1*^−/−^ mice were either infected i.v. with *E. coli* at a dose of 2.5 × 10^7^ colony forming units (CFU)/g of body weight or injected with PBS. Mice were euthanized after 24 h, and livers were harvested. (**A**) Phospho-p38 levels in the livers of control and *E. coli*-infected mice. Parts of the livers were homogenized to extract soluble proteins. Protein samples (40 µg) from distinct animals were resolved to detect phospho-p38 by Western blotting, using a polyclonal antibody against phospho-p38. The membranes were stripped and reblotted with p38 antibody to verify comparable loading. Images shown in the left panel are representative results. Bar graph in the right panel represent quantification of phosphor-p38 (p-p38) levels in each groups (*n* = 4). Groups marked with distinct letters above the bars indicate significant differences (*p* < 0.05, two-way Analysis of variance (ANOVA)); (**B**) Liver triglyceride; (**C**) Total liver lipid; (**D**) Liver cholesterol. Values in B-D represent means ± SE from 10 different animals, and data were analyzed by two-way ANOVA with Tukey post-test to evaluate main and interactive effects. NS: not significant.

Since p38 is a preferred substrate of Mkp-1 [31] and plays a critical role in the regulation of the inflammatory response [25,30,32,33], we assessed p38 activity in the livers of *Mkp-1*^+/+^ and *Mkp-1*^−/−^ mice both before and after *E. coli* infection (Figure 1A). In the absence of infection liver p38 activity was higher in the *Mkp-1*^−/−^ mice than in the *Mkp-1*^+/+^ mice. In *E. coli*-infected wildtype mice, liver p38 activity became virtually undetectable 24 h post infection. In contrast, liver p38 activity remained high in *E. coli*-infected *Mkp-1*^−/−^ mice. Quantification of p38 activity in the four groups further highlights the essential role of Mkp-1 both in the regulation of basal p38 activity in uninfected mice and particularly in the deactivation of p38 following *E. coli* infection (Figure 1A, right panel).

**Figure 2:**
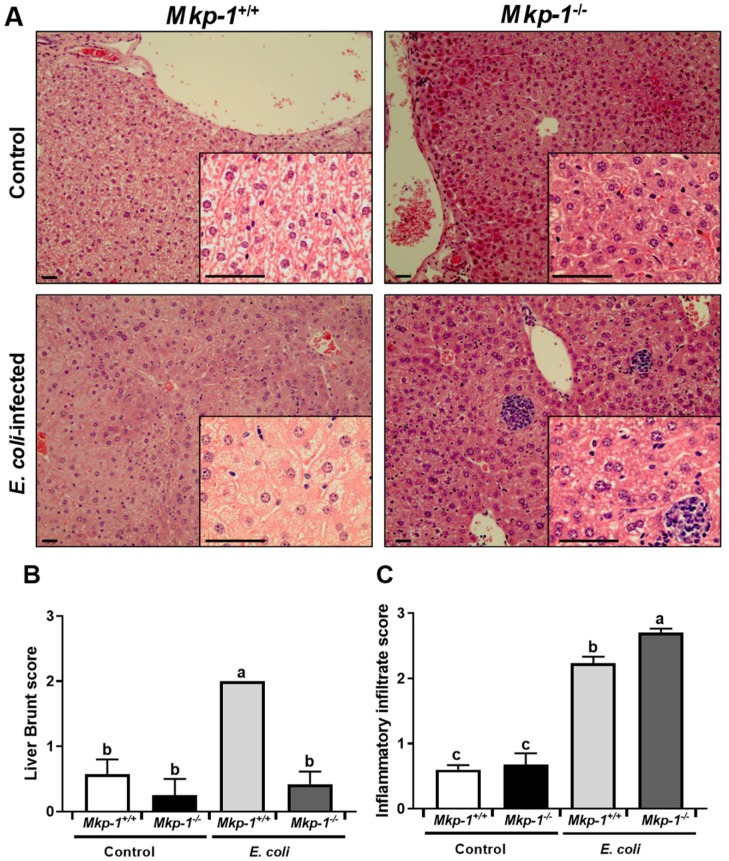
Liver histology of *Mkp-1*^+/+^ and *Mkp-1*^−/−^ mice before and after *E. coli* infection. Control and *E. coli*-infected mice (2.5 × 10^7^ CFU/g of body weight, i.v.) were euthanized 24 h post infection. Small portions of the livers were excised and fixed in formalin, paraffinized, and sectioned for histological assessment. Liver lipid level was scored according to Brunt steatosis scoring system. (**A**) Histology of livers from control and *E. coli*-infected mice. Images are representative H&E-stained liver sections. Outset magnification ×100; inset ×400; (**B**) Liver lipid score evaluated by the Brunt steatosis scoring system; (**C**) Hepatic inflammatory infiltrate score. Hepatic inflammatory infiltrate score was evaluated by counting neutrophils in 20 randomly selected optical fields. Two-way ANOVA was conducted to detect group differences. Groups without a common superscript were significantly different (*p* < 0.05).

We have previously shown that *Mkp-1*^+/+^ mice but not *Mkp-1*^−/−^ mice developed hyperglyceridemia in response to *E. coli* infection [10]. To understand how Mkp-1 deficiency and sepsis affect liver lipid contents, we measured triglyceride, total lipid, and cholesterol levels in the livers. Consistent with a previous report [34], uninfected *Mkp-1*^−/−^ mice had lower liver triglyceride than uninfected *Mkp-1*^+/+^ mice (Figure 1B). Surprisingly, *E. coli* infection significantly increased total liver triglyceride levels in *Mkp-1*^+/+^ mice, but not in *Mkp-1*^−/−^ mice (Figure 1C). *E. coli* infection also increased total liver lipid content in the *Mkp-1*^+/+^ mice, but not in *Mkp-1*^−/−^ mice (Figure 1C), while liver cholesterol content did not differ between groups (Figure 1D). The differences in liver lipid contents were corroborated by histology analyses (Figure 2A). Diffused hepatocellular swelling and clearing, an indication of hepatic glycogen deposition, were seen in both un-infected *Mkp-1*^+/+^ and *Mkp-1*^−/−^ mice, although the hepatocellular swelling and clearing were more prevalent in *Mkp-1*^+/+^ mice than in *Mkp-1*^−/−^ mice (Figure 2A, Top row). Upon *E. coli* infection, dramatic differences were seen in the histology between the *Mkp-1*^+/+^ and *Mkp-1*^−/−^ livers (Figure 2A, bottom row). Histological examination revealed moderate to marked, centrilobular to midzonal, hepatocyte vacuolation (often referred to as hepatocyte ballooning) in the livers of *E. coli*-infected *Mkp-1*^+/+^ mice, indicating hepatic lipidosis. Additionally, multifocal-random moderate to marked necrosuppurative hepatitis with rare intralesional rod-shaped bacteria were seen throughout the *Mkp-1*^+/+^ liver sections. Furthermore, in *Mkp-1*^+/+^ liver sections numerous neutrophils and necrotic cellular debris were randomly distributed throughout hepatic parenchyma and occasionally formed abscesses. In contrast, *E. coli*-infected *Mkp-1*^−/−^ livers exhibited marked midzonal hepatocellular swelling and clearing, suggesting considerable glycogen levels in these mice. Additionally, multifocal-random and marked necrosuppurative hepatitis was seen in the *Mkp-1*^−/−^ liver sections. Hepatic abscesses in the *Mkp-1*^−/−^ livers were predominantly comprised of numerous bacterial rods intermixed with cellular necrotic debris and neutrophils. Quantitation of hepatocyte ballooning using the Brunt liver steatosis scores system confirmed more lipid droplets in liver sections of uninfected *Mkp-1*^+/+^ mice that in those of uninfected *Mkp-1*^−/−^ mice (Figure 2B). *E. coli* infection further enhanced hepatocyte lipidosis in *Mkp-1*^+/+^ mice but not in *Mkp-1*^−/−^ mice. As expected, *E. coli* infection resulted in a prominent increase in inflammatory infiltrate score, which is further exacerbated in *Mkp-1*^−/−^ mice (Figure 2C).

### 2.2. The Impact of Mkp-1 Deficiency and E. coli Infection on Global Liver Gene Expression Profile

**Figure 3:**
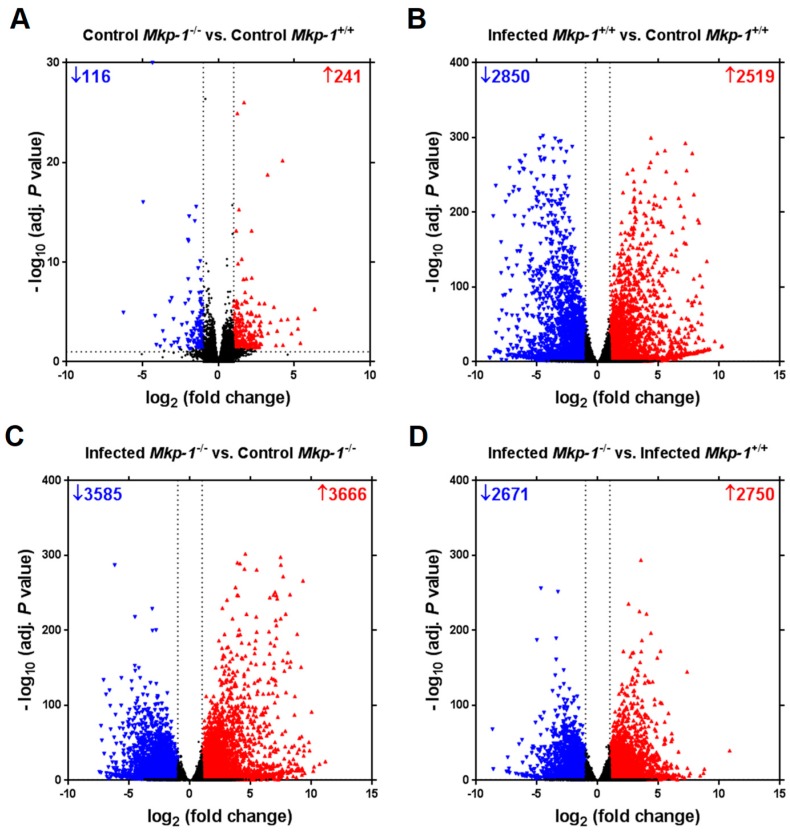
Differentially expressed genes in *Mkp-1*^+/+^ and *Mkp-1*^−/−^ mice before and following *E. coli* infection. *Mkp-1*^+/+^ and *Mkp-1*^−/−^ mice were either infected i.v. with *E. coli* at a dose of 2.5 × 10^7^ CFU/g of body weight or injected with PBS (controls). Mice were euthanized after 24 h, and total RNA was isolated from the livers of four mice using Trizol for RNA-seq analyses. Volcano plots show the extent of differentially expressed gene (adjusted *p* value < 0.05, absolute value of log_2_ fold change 2) in each of four comparisons: control *Mkp-1*^+/+^ vs. control *Mkp-1*^−/−^ (**A**), infected *Mkp-1*^+/+^ vs. control *Mkp-1*^+/+^ (**B**), infected *Mkp-1*^−/−^ vs. control *Mkp-1*^−/−^ (**C**), and infected *Mkp-1*^−/−^ vs. infected *Mkp-1*^+/+^ (**D**).

To explore the mechanisms of lipid dysregulation caused by *Mkp-1* deficiency and sepsis, RNA-seq was conducted using hepatic RNA extracts. Without *E. coli* infection, *Mkp-1* deficiency increased the mRNA expression of 241 genes and decreased the mRNA expression of 116 genes (Figure 3A). In *Mkp-1*^+/+^ mice, *E. coli* infection enhanced the expression of 2519 genes and lowered the expression of 2850 genes (Figure 3B). In contrast, *E. coli* infection caused the upregulation of 3666 genes and downregulation of 3585 genes in *Mkp-1*^−/−^ mice (Figure 3C). Impressively, compared to *E. coli*-infected wildtype mice, 2750 genes were upregulated and 2671 genes were downregulated in *E. coli*-infected *Mkp-1*^−/−^ mice, suggesting profound exacerbation of the host transcriptional responses in the livers of *Mkp-1*^−/−^ mice (Figure 3D).

**Figure 4:**
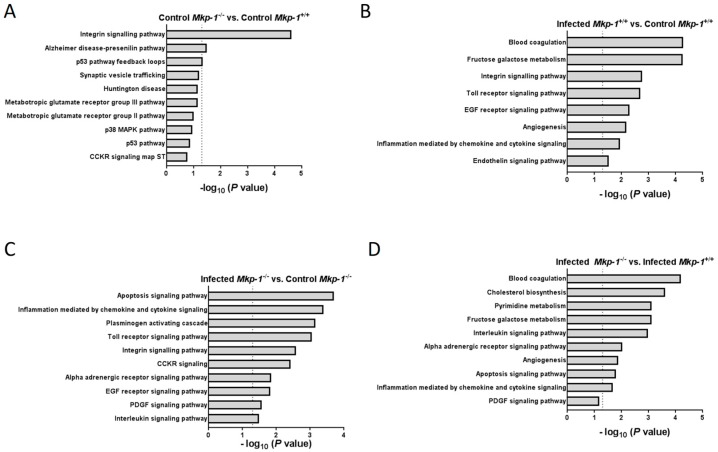
Pathway over-representation analysis for differentially expressed transcripts in control and *E. coli*-infected *Mkp-1*^+/+^ and *Mkp-1*^−/−^ mice. RNA-seq data were analyzed with the Panther pathway classification system. Bar plots depict overrepresented Panther pathways affected by Mkp-1 knockout and/or *E. coli* infection in the following comparisons: control *Mkp-1*^−/−^ vs. control *Mkp-1*^+/+^ (**A**), *E. coli*-infected *Mkp-1*^+/+^ vs. control *Mkp-1*^+/+^ (**B**), *E. coli*-infected *Mkp-1*^−/−^ vs. control *Mkp-1*^−/−^ (**C**), and *E. coli*-infected *Mkp-1*^−/−^ vs. *E. coli*-infected *Mkp-1*^+/+^ (**D**). Dotted lines indicate *p* = 0.05 on the –log_10_ scale.

We also categorized the differentially expressed genes in the liver, by Panther pathway analysis. In the absence of infection, genes of several pathways were differentially expressed in the livers of *Mkp-1*^−/−^ mice relative to those of wildtype mice, including the integrin signaling pathway, the p53 pathway, and the p38 pathway (Figure 4A). Consistent with the idea that *E. coli* infection elicits a landscape change in inflammatory gene expression, upon *E. coli* infection genes in the Toll-like receptor (TLR) pathway and inflammatory cytokine and chemokine pathways were substantially altered in the wildtype mice (Figure 4B). In the *Mkp-1* knockout mice the inflammatory response, indicated by alteration in the chemokine and cytokine signaling pathways, TLR pathways, as well as interleukin signaling pathways, were dramatically enhanced after *E. coli* infection (Figure 4C). Moreover, blood coagulation, cholesterol biosynthesis, and inflammatory cytokine and chemokine pathways were more robustly altered by *Mkp-1* deficiency in the *E. coli*-infected mice (Figure 4D). Interestingly, the cholesterol biosynthesis as well as blood coagulation genes were significantly altered upon *Mkp-1* deletion in the *E. coli*-infected mice.

**Figure 5:**
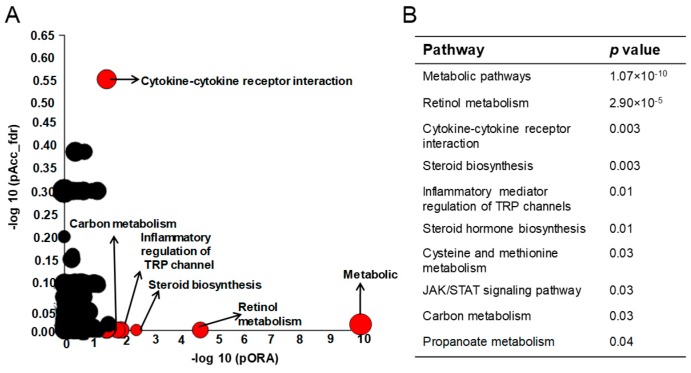
Differential affected pathways in the livers of *Mkp-1*^+/+^ and *Mkp-1*^−/−^ mice by *E. coli* infection. The RNA-seq data were analyzed using the iPathwayGuide analytic platform to identify the pathways differentially modulated in *Mkp-1*^+/+^ and *Mkp-1*^−/−^ mice by *E. coli* infection. (**A**) Pathways differentially modulated in *Mkp-1*^+/+^ and *Mkp-1*^−/−^ mice. Pathway analysis was done on the differentially expressed genes (>2-fold change either direction) using iPathwayGuide analysis tool. Each pathway is represented by a single dot in the graph for over-representation on the horizontal axis (pORA) and the perturbation on the vertical axis (pAcc), with the size of the dot proportional to the number of genes differentially modulated in that pathway. Differentially modulated pathways (FDR < 5%) were shown in red and pathways that did not reach statistical significance were shown in black. Note: In both axis, *p*-values are shown on the −log_10_ scale; (**B**) The top 10 pathways differentially modulated in *Mkp-1^+/+^* and *Mkp-1^−/−^* mice by *E. coli* infection according to the pORA values.

We also analyzed the RNA-seq data using DESeq2 algorithm to identify the pathways differentially affected in *Mkp-1*^+/+^ and *Mkp-1*^−/−^ mice after *E. coli* infection (Figure 5A). Among the pathways that were preferentially affected by *E. coli* infection in the two strain of mice were various metabolic processes, including retinol metabolism, steroid synthesis, and carbon metabolism (Figure 5B), suggesting an important function of Mkp-1 in broad metabolic functions.

### 2.3. E. coli Infection Caused a Major Shift in Gene Expression of the Fatty Acid and Glucose Metabolic Programs and Mkp-1 Deficiency Disrupts This Shift

**Figure 6:**
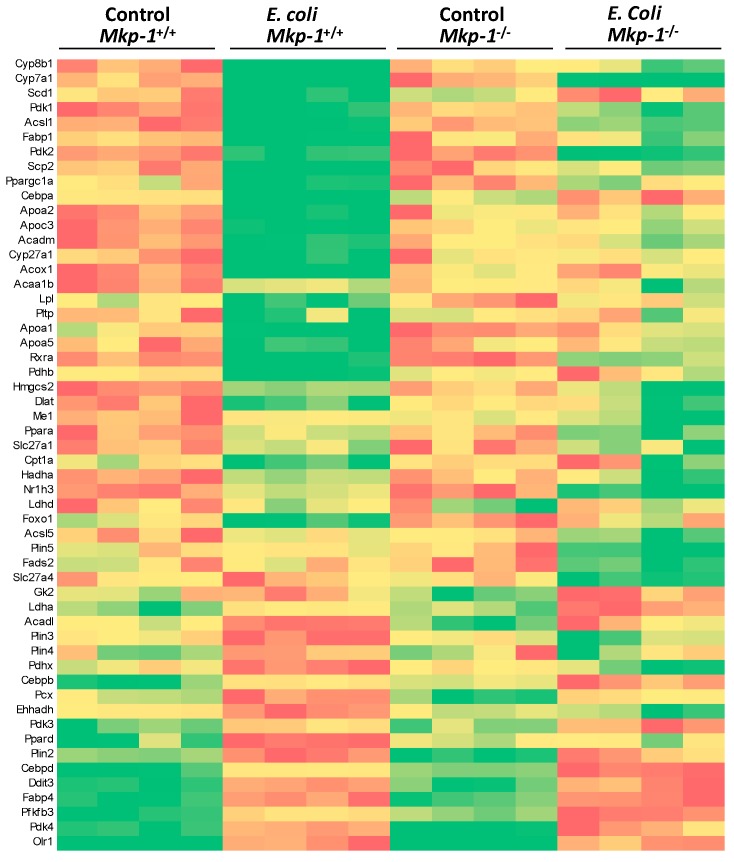
Expression of fatty acid metabolism genes significantly altered by *E. coli* infection and/or *Mkp-1* knockout. Heat map showing relative changes in expression of select transcripts in individual mice as assessed using RNA-seq. The color gradient ranges from red (highest levels of expression) to green (lowest expression levels), with yellow representing intermediate levels. Note: Each lane represents a different animal, and only significantly affected genes were shown in the heat map (*p* < 0.05, Student’s *t*-test, *n* = 4).

Because of the striking differences in the triglyceride contents between *Mkp-1*^+/+^ and *Mkp-1*^−/−^ mice, particularly after *E. coli* infection, we focused on the proteins involved in triglyceride and fatty acid metabolisms. We found that fatty acid metabolism and hepatic glycolysis/gluconeogenesis pathways were profoundly altered by both *Mkp-1* deficiency and *E. coli* infection (Figure 6). The heat map presented in Figure 6 depicts the expression levels of carbohydrate metabolism genes significantly altered by *E. coli* infection and/or *Mkp-1* knockout. A number of genes involved in fatty acid transport, such as fatty acid-binding protein 1 (Fabp1) and four apolipoproteins (Apoa1, 2, 5 and Apoc3), were downregulated by *E. coli* infection in *Mkp-1*^+/+^ mice. Additionally, *E. coli* infection also attenuated the expression of genes involved in fatty acid synthesis and utilization, such as stearoyl-CoA desaturase 1 (Scd1), long chain fatty acid-CoA ligase 1 (Acsl1), acyl-CoA thioesterase (Acot) 1 and 4, peroxisome proliferator-activated receptor (PPAR) γ coactivator 1-α and β (Ppargc1a and b), Forkhead box protein O1 (Foxo1), acetyl-CoA acyltransferase 1 (Acaa1b), and CCAAT/enhancer-binding protein (Cebp) α (Cebpa) in *Mkp-1*^+/+^ mice. Furthermore, a number of perilipin (Plin) genes, including Plin 2, 3, and 4 were upregulated in *Mkp-1*^+/+^ mice. Interestingly, many genes involved in glycolysis were upregulated by *E. coli* infection in both *Mkp-1*^+/+^ and *Mkp-1*^−/−^ mice, including pyruvate dehydrogenase kinase (Pdk) 3/4, and 6-phosphofructo-2-kinase/fructose-2,6-biphosphatase 3 (Pfkfb3). Most importantly, *E. coli* infection-induced changes of the majority of these genes were markedly altered by *Mkp-1* deficiency. The profound changes in the transcriptome of the lipid and carbohydrate metabolic pathways suggest that a major shift in carbon/energy metabolism occurred in wildtype mice upon *E. coli* infection and that the Mkp-1 protein is required for this metabolic shift.

### 2.4. E. coli Infection Lowers the Expression of Mammalian Target of Rapamycin (mTOR) and Lipogenic Genes

**Figure 7:**
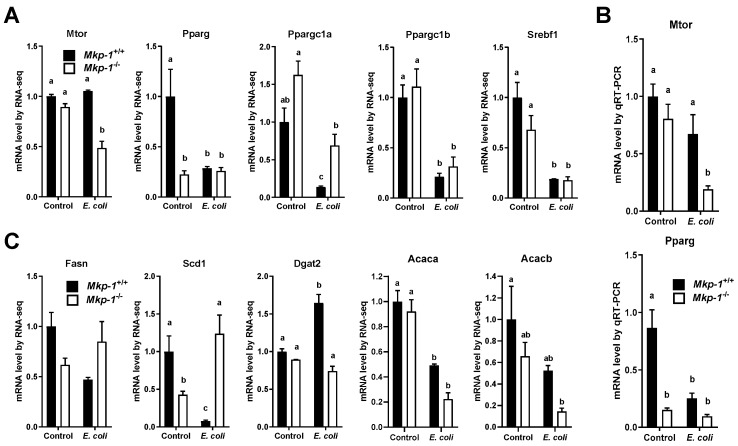
Hepatic mRNA expression of lipogenesis genes before and after *E. coli* infection. *Mkp-1*^+/+^ and *Mkp-1*^−/−^ mice were either infected i.v. with *E. coli* at a dose of 2.5 × 10^7^ CFU/g of body weight or injected with PBS. Mice were euthanized after 24 h, and total RNA was isolated from the livers using Trizol. mRNA expression for different genes was assessed based on the RNA-seq data set, or quantified via qRT-PCR. Expression in un-infected *Mkp-1*^+/+^ mice was set as 1. Values represent means ± S.E. from 4 animals for RNA-seq and 4–7 animals for qRT-PCR in each group. (**A**) Expression levels of lipogenic regulator genes based on RNA-seq; (**B**) Expression levels of Mtor and Pparg based on qRT-PCR; (**C**) Expression levels of lipogenic genes based on RNA-seq. Values in the graphs were compared by two-way ANOVA. Groups marked with distinct letters above the bars indicate significant differences (*p* < 0.05).

The hepatic mTOR/Akt signaling promotes hepatic lipid biosynthesis by activating SREBP-1 and increasing PPARγ expression [35]. RNA-seq data demonstrated that infected *Mkp-1*^−/−^ mice had downregulated mTOR (designated as Mtor for mouse) expression relative to infected *Mkp-1*^+/+^ mice, although the expression levels in uninfected mice were similar in *Mkp-1*^+/+^ and *Mkp-1*^−/−^ mice (Figure 7A). The expression of Pparg (the murine ortholog of PPARγ) was lower in uninfected *Mkp-1*^−/−^ mice than in uninfected *Mkp-1*^+/+^ mice, although *E. coli* infection resulted in a decrease in Pparg expression in both groups. The expression of three other lipogenic regulators, Ppargc1a (murine ortholog of PPARγ coactivator (PGC)-1α)), Ppargc1b (murine ortholog of PGC-1β), and Srebf1 (murine ortholog of SREBP-1), were similar between *Mkp-1*^+/+^ and *Mkp-1*^−/−^ mice. *E. coli* infection resulted in a decrease in their expression in both *Mkp-1*^+/+^ and *Mkp-1*^−/−^ groups. The differences observed by RNA-seq for both Mtor and Pparg expression were confirmed by quantitative reverse transcription PCR (qRT-PCR) (Figure 7B). Additionally, RNA-seq also identifies some differences in the expression of five lipogenic genes, Fasn, Scd1, acetyl-CoA carboxylase α (designated as Acaca for the murine ortholog), acetyl-CoA carboxylase β (designated as Acacb for the murine orthoolog), and diglyceride acyltransferase 2 (Dgat2) (Figure 7C). In *Mkp-1*^+/+^ mice, *E. coli* infection downregulated the expression of Fasn and Scd1 (Figure 7C), but modestly increased the mRNA expression of Dgat2, an enzyme responsible for synthesis of triglyceride from fatty acid and glycerol [36,37]. In contrast, Scd1 expression was enhanced in *Mkp-1*^−/−^ mice following *E. coli* infection. Acetyl-CoA carboxylase, particularly Acaca, catalyzes the carboxylation of acetyl-CoA to malonyl-CoA, the rate-limiting step in fatty acid synthesis. Although the levels of liver Acaca mRNA in *Mkp-1*^+/+^ and *Mkp-1*^−/−^ mice were similar, *E. coli* infection led to a decrease in liver Acaca mRNA expression in both groups (Figure 7C). Unlike Acaca which primarily regulates fatty acid synthesis, Acacb plays an important role in fatty acid oxidation. The expression levels of Acacb mRNA did not substantially differ except between un-infected *Mkp-1*^+/+^ and *E. coli*-infected *Mkp-1*^−/−^ mice (Figure 7C).

**Figure 8:**
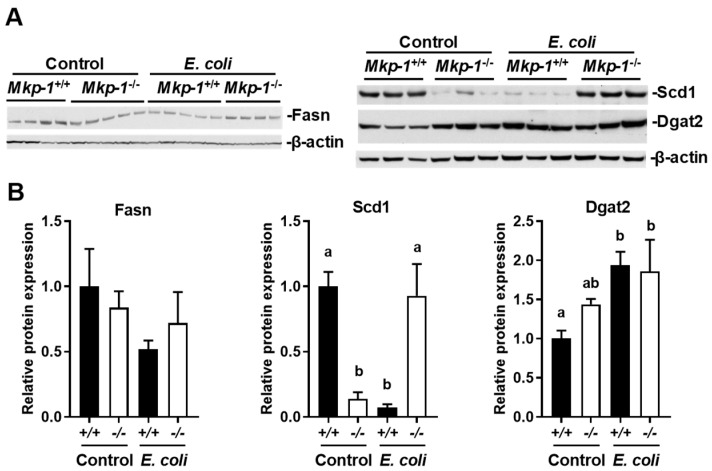
Levels of liver lipogenic proteins in control and *E. coli*-infected mice. Control and *E. coli*-infected mice (2.5 × 10^7^ CFU/g of body weight, i.v.) were euthanized 24 h post infection. (**A**) Representative results of Western blot analyses. Liver protein extracts from control or *E. coli*-infected *Mkp-1*^+/+^ and *Mkp-1*^−/−^ mice were subjected to Western blotting with the indicated antibodies. The housekeeping protein β-actin was used as a control for normalization of protein loading; (**B**) Quantification of protein expression. The images were scanned by densitometry and expression levels normalized to β-actin. The expression level in control *Mkp-1*^+/+^ mice was set as 1. The results were analyzed by two-way ANOVA. Groups marked with distinct letters above the bars indicate significant differences (*p* < 0.05).

To assess the levels of the lipogenic proteins in the livers, Western blot analyses were performed using liver homogenates (Figure 8). Fasn protein levels in *E. coli*-infected *Mkp-1*^+/+^ mice appeared lower although the difference was not statistically significant (Figure 8). Scd protein levels mirrored the differences in Scd1 mRNA levels. In the absence of *E. coli* infection, Scd1 protein levels were substantially lower in *Mkp-1*^−/−^ mice than in *Mkp-1*^+/+^ mice. While liver Scd1 protein levels dramatically plummeted in *Mkp-1*^+/+^ mice following *E. coli* infection, liver Scd1 protein levels were significantly increased in *Mkp-1*^−/−^ mice. Dgat2 protein levels were significantly increased in *Mkp-1*^+/+^ mice following *E. coli* infection, but did not significantly change in *Mkp-1*^−/−^ mice.

### 2.5. E. coli Infection Increased the Expression of Genes Involved in Liver Fatty Acid Uptake, and Lowered the Expression of Genes Involved in Mitochondrial and Peroxisomal Fatty Acid Oxidation

**Figure 9:**
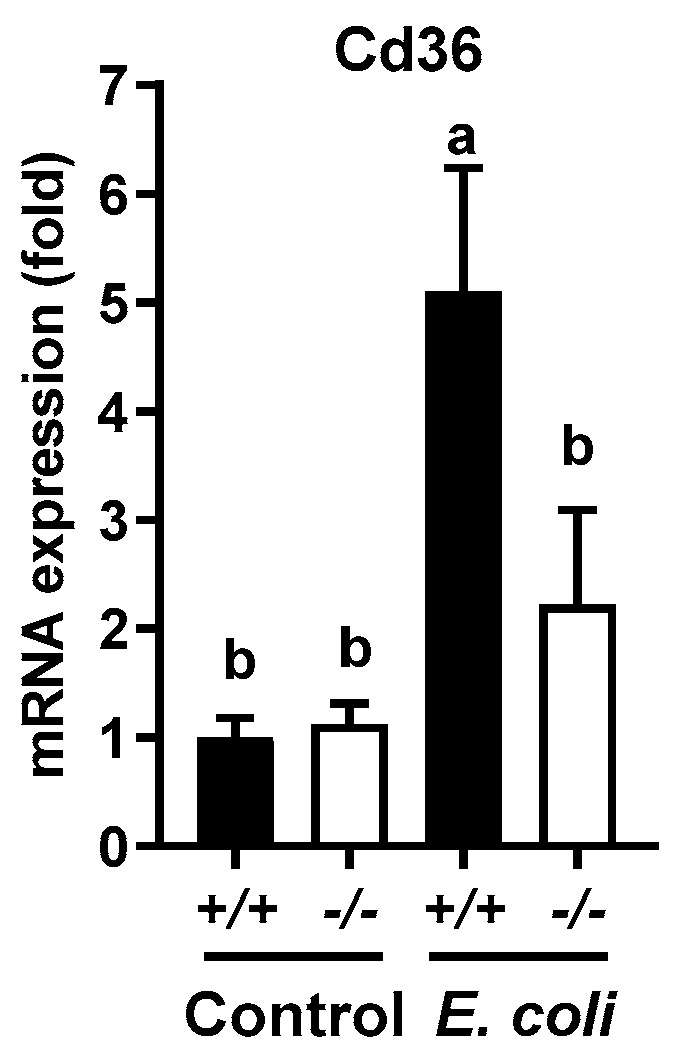
Liver mRNA expression levels of Cd36 in control and *E. coli*-infected mice. Control and *E. coli*-infected mice (2.5 × 10^7^ CFU/g of body weight, i.v.) were euthanized 24 h post infection. Total RNA was isolated from the livers using Trizol. Cd36 mRNA levels were assessed via qRT-PCR. Expression in un-infected *Mkp-1*^+/+^ mice was set as 1. Values represent means ± S.E. from 4–7 animals in each group. The results were analyzed by two-way ANOVA. Groups marked with distinct letters above the bars indicate significant differences (*p* < 0.05).

Fatty acid uptake is an important mechanism for lipid accumulation in the liver. In response to the decline of blood glucose level, fat tissues release fatty acids through lipolysis. Cluster of differentiation 36 (Cd36) plays an important role in fatty acid uptake from the blood stream for lipogenesis in the liver [38]. To understand the mechanism underlying the differential effects of *E. coli* infection on liver and blood triglyceride contents of *Mkp-1*^+/+^ and *Mkp-1*^−/−^ mice, we measured Cd36 levels by qRT-PCR (Figure 9). Liver Cd36 mRNA levels were similar between control *Mkp-1*^+/+^ and *Mkp-1*^−/−^ mice, and *E. coli* infection caused a significant increase in Cd36 mRNA levels in *Mkp-1*^+/+^ mice but had little effect on Cd36 mRNA levels in *Mkp-1*^−/−^ mice.

**Figure 10:**
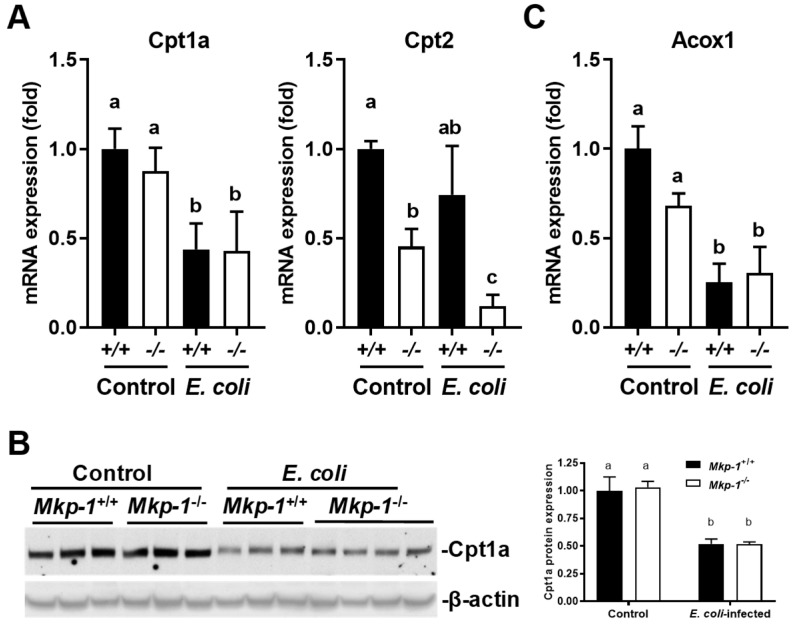
Hepatic expression of fatty acid β-oxidation proteins or genes before and after *E. coli* infection. *Mkp-1*^+/+^ and *Mkp-1*^−/−^ mice were either infected i.v. with *E. coli* at a dose of 2.5 × 10^7^ CFU/g of body weight or injected with PBS. Livers were harvested 24 h post infection to isolate total RNA or protein. The mRNA and protein levels were assessed by qRT-PCR or Western blot analyses. (**A**) mRNA levels of fatty acid oxidation proteins assessed by qRT-PCR; (**B**) Representative Western blot of Cpt1a protein. Forty µg of protein for each sample was used for Western blot analysis. Each lane represents an individual animal. The membrane was stripped and reblotted with β-actin antibody to verify comparable loading. The densities of the bands were determined by densitometry, normalized to β-actin levels, and the relative expression level of Cpt1a protein in each group was depicted as means ± SE in the graph on the right (*n* = 3–4 mice per group); (**C**) Acox1 mRNA levels assessed by qRT-PCR. Expression in un-infected *Mkp-1*^+/+^ mice was set as 1. Values represent means ± S.E. of 3–5 different animals in each group. Values were compared by two-way ANOVA. Groups marked with distinct letters above the bars indicate significant differences (*p* < 0.05).

We also assessed the expression of genes involved in fatty acid β-oxidation through qRT-PCR (Figure 10A). Carnitine palmitoyltransferase I and II (Cpt1a and Cpt2) are essential enzymes for β-oxidation of long chain fatty acid in mitochondria [39,40], while acyl-CoA oxidase 1 (Acox1) mediates fatty acid β-oxidation in peroxisome [39,41]. Cpt1a mRNA expression was significantly decreased in both *Mkp-1*^+/+^ and *Mkp-1*^−/−^ mice upon *E. coli* infection, although expression levels in control conditions were similar in the two genotypes of mice. Liver Cpt2 mRNA expression levels were lower in control *Mkp-1*^−/−^ mice than in control *Mkp-1*^+/+^ mice. *E. coli* infection decreased Cpt2 mRNA levels in *Mkp-1*^−/−^ mice but had little effect in *Mkp-1*^+/+^ mice. Acox1 mRNA levels were similar in *Mkp-1*^+/+^ and *Mkp-1*^−/−^ mice, and were decreased in both genotypes upon *E. coli* infection. Cpt1a protein levels, detected by Western blotting, were similar in un-infected *Mkp-1*^+/+^ and *Mkp-1*^−/−^ mice, and *E. coli* infection caused a significant decrease in both *Mkp-1*^+/+^ and *Mkp-1*^−/−^ mice (Figure 10B). Taken together, these results suggest that fatty acid oxidation in the liver was decreased with *E. coli* infection while fatty acid uptake was increased in *Mkp-1*^+/+^ but not in *Mkp-1*^−/−^ mice.

### 2.6. Effects of E. coli Infection and Mkp-1 Deficiency on the Expression of Phosphoenolpyruvate Carboxykinase 1 (Pck1) Protein

**Figure 11:**
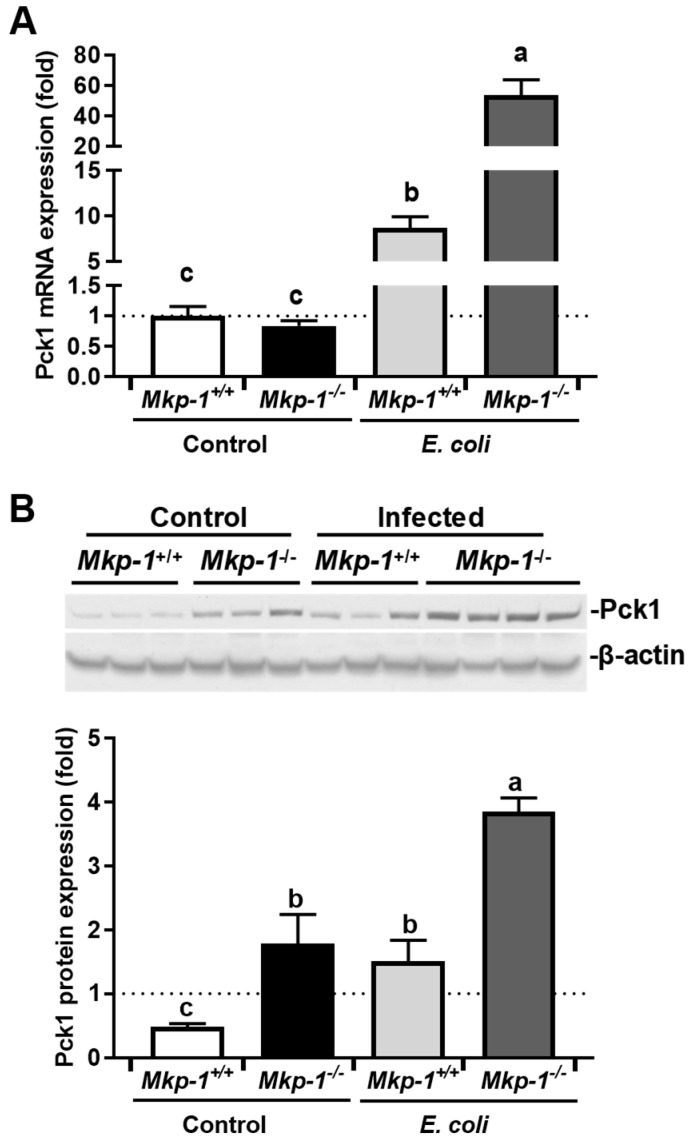
Hepatic PCK1 expression in *Mkp-1*^+/+^ and *Mkp-1*^−/−^ mice before and after *E. coli* infection. *Mkp-1*^+/+^ and *Mkp-1*^−/−^ mice were either infected i.v. with *E. coli* at a dose of 2.5 × 10^7^ CFU/g of body weight or injected with PBS. Mice were euthanized after 24 h to extract total RNA and protein. (**A**) Pck1 mRNA expression levels were quantitated via qRT-PCR. Expression in un-infected *Mkp-1*^+/+^ mice was set as 1. Values represent means ± S.E. from 4–5 different animals in each group; (**B**) Liver Pck1 protein levels. Liver protein extracts from control or *E. coli*-infected *Mkp-1*^+/+^ and *Mkp-1*^−/−^ mice were subjected to Western blotting with a mouse monoclonal antibody against Pck1. Each lane represents an individual animal. The membranes were stripped and then used to blotting with β-actin antibody. Representative results from Western blot analyses are shown. The densities of the bands were determined by densitometry, and normalized to β-actin levels, and the relative expression levels of Pck1 protein in each group were depicted as means ± SE in the graph below. Values were compared by two-way ANOVA. Groups marked with distinct letters above the bars indicate significant differences (*p* < 0.05).

The cytosolic Pck1 protein, often referred to as phosphoenolpyruvate carboxykinase-cytosolic isoform (PEPCK-c), is an important regulator in gluconeogenesis [42,43,44]. Hepatic Pck1 facilitates gluconeogenesis by synthesizing phosphoenolpyruvate from oxaloacetate [45]. Previously, it has been shown that liver Pck1 expression is enhanced in *Mkp-1*^−/−^ mice in control conditions [46]. Our RNA-seq analysis indicates that liver Pck1 mRNA levels were significantly increased in both *Mkp-1*^+/+^ and *Mkp-1*^−/−^ mice following *E. coli* infection. Although the basal Pck1 mRNA levels in *Mkp-1*^+/+^ and *Mkp-1*^−/−^ mice were similar (Figure 11A), liver Pck1 protein levels in control *Mkp-1*^−/−^ mice were significantly higher than those in control *Mkp-1*^+/+^ mice (Figure 11B). *E. coli* infection further enhanced the expression of Pck1 protein in both *Mkp-1*^+/+^ and *Mkp-1*^−/−^ mice (Figure 11B). The enhanced expression of Pck1 suggests that both *Mkp-1* knockout and *E. coli* infection stimulate gluconeogenesis.

## 3. Discussion

We have previously shown that *Mkp-1*^−/−^ mice exhibited significant increases in both inflammatory cytokine production and mortality after *E. coli* infection relative to *Mkp-1*^+/+^ mice [10]. Our earlier studies have also shown that *E. coli* infection triggers a dramatic increase in blood triglyceride levels in *Mkp-1*^+/+^ mice but not in *Mkp-1*^−/−^ mice [10], suggesting profound alterations in lipid metabolism in the *Mkp-1*^−/−^ mice following *E. coli* infection. To understand the role of Mkp-1 in the regulation of metabolism during sepsis, we analyzed the lipid contents and global gene expression profiles in the livers of *Mkp-1*^+/+^ and *Mkp-1*^−/−^ mice either in control conditions or after *E. coli* infection. Here we report that *E. coli* infection increased liver lipid content in *Mkp-1*^+/+^ mice (Figure 1), indicating that hyperlipidemia after *E. coli* infection was not the result of depletion of hepatic lipid stores. The increase in liver lipid content in *Mkp-1*^+/+^ mice was supported by hepatocyte ballooning after *E. coli* infection (Figure 2). RNA-seq analysis of the liver tissues detected a profound difference in gene expression profiles between wildtype and *Mkp-1*^−/−^ mice in the liver following *E. coli* infection (Figure 3). Remarkably, over 5000 genes (>20% of all murine genes) exhibited a >2-fold difference in expression levels between the two groups of mice after *E. coli* infection (Figure 3D), highlighting the critical role of Mkp-1 in the liver response to sepsis. Interestingly, *E. coli* infection caused profound changes in the expression of many genes involved in lipid metabolism, including fatty acid uptake, utilization, and synthesis in *Mkp-1*^+/+^ mice (Figure 6). However, in *Mkp-1^−/−^* mice the *E. coli* infection-induced changes in lipid metabolism-related genes were profoundly disrupted. In other words, unlike in the wildtype livers, in the absence of Mkp-1 the livers were unable to adjust their lipid metabolic program (Figure 6).

### 3.1. The Critical Role of Mkp-1 as a p38 Regulator in the Liver

Consistent with the notion that p38 is the preferred substrate of Mkp-1, we found that base line p38 activity was higher in uninfected *Mkp-1*^−/−^ livers than in *Mkp-1*^+/+^ livers (Figure 1A). This indicates that Mkp-1 is critical in the control of p38 activity in the liver under normal conditions. Interestingly, in pre-clinical studies and clinical trials a common side effect of the p38 inhibitors is hepatotoxicity [47], suggesting that base line p38 activity is required for protection of the liver. Elevated base line p38 activity could be directly or indirectly implicated in the changes seen in the metabolic pathways and transcriptome. Remarkably, we found that liver p38 activity is dramatically decreased 24 h after *E. coli* infection in *Mkp-1*^+/+^ mice (Figure 1A), while it remained high in *Mkp-1*^−/−^ mice. Considering that *Mkp-1* is a gene highly inducible by extracellular stimuli [29,32,33,48,49], a systemic *E. coli* infection likely enhanced the expression of *Mkp-1* in the liver of the *Mkp-1*^+/+^ leading to the de-phosphorylation of p38. In the absence of the *Mkp-1* gene, it is not surprising that p38 remained high in the livers of the *E. coli*-infected *Mkp-1*^−/−^ mice. Since p38 plays an important role in inflammatory responses [50], persistently high p38 activity in the livers of *E. coli*-infected *Mkp-1*^−/−^ mice provides a plausible explanation for the differential expression of genes involved in inflammation (Figure 4 and Figure 5).

### 3.2. Mkp-1 and Hyperlipidemia of Sepsis

In a prior study we reported that *E. coli* infection caused a 13-fold increase in blood triglyceride levels in *Mkp-1*^+/+^ mice, but not the *Mkp-1*^−/−^ mice [10]. This is consistent with the work of others reporting that bacterial endotoxin causes ‘lipemia of sepsis’ by increasing VLDL-mediated triglyceride release from hepatocytes [14], and by limiting lipoprotein lipase-mediated VLDL clearance in peripheral tissues [17,18,51]. Lipoproteins, such as triglyceride-rich VLDL, not only provide fuel to the host to fight against bacterial infection, but also sequester and neutralize endotoxin [14,52]. Therefore, hypertriglyceridemia during sepsis may be an adaptive host response to bacterial infections. Our studies indicate that *E. coli* infection induced increases in not only blood triglyceride [10] but also liver triglyceride content (Figure 1) in a Mkp-1-dependent manner, raised a strong possibility that the liver actually accelerates glyceride synthesis during sepsis. While it is unclear how the wildtype mice manage to increase blood triglyceride levels, while also increasing liver triglyceride contents, we can speculate. In our experimental setting we observed that *Mkp-1*^+/+^ mice usually stop feeding 2–3 h after *E. coli* infection, which is likely the result of an acute phase response and cytokine storm. Infected mice usually abstain from feeding in the first few days then then resume feeding as they recover. The septic mice were sacrificed at 24 h for the measurement of blood and liver lipid contents. Thus, the acute increase in triglyceride levels in blood and liver of *E. coli*-infected *Mkp-1*^+/+^ mice were unlikely due to increased consumption of food [53]. Instead, the increased blood triglyceride content is probably due to increased catabolism of adipose and muscle tissues, as TNF-α produced following *E. coli* infection has been shown to suppress lipogenesis and enhance lipolysis in these tissues [54,55]. We speculate that increased liver triglyceride following infection in the wildtype mice is more likely the result of enhanced hepatic lipogenesis from fatty acids and glycerol taken up from the blood rather than from fatty acids synthesized de novo from acetyl-CoA in the liver for the following reasons. First, a number of lipogenic genes involved in triglyceride synthesis from acetyl-CoA, including Pparg (murine ortholog of PPARγ), Ppargc1a/b (murine ortholog of PGC1α/β), Srebf1, Fasn, Scd1, and Acaca that catalyzes the rate-limiting step in fatty acid synthesis, were substantially downregulated (Figure 6 and Figure 7). Second, the expression of Dgat2, the liver enzyme responsible for triglyceride synthesis from fatty acids and glyceride [56], was increased in *Mkp-1*^+/+^ mice after *E. coli* infection (Figure 7C and Figure 8B). Finally, mRNA expression of Cd36, the liver protein mediating fatty acid uptake [38], is markedly upregulated in *Mkp-1*^+/+^ mice after *E. coli* infection. Fatty acids taken up from the blood can be converted in the liver to triglyceride by Dgat2 [56]. We think that the liver likely synthesizes triglyceride for consumption by other organs, because both the mitochondrial fatty acid β-oxidation-related Cpt1a protein [39,40]) and peroxisomal fatty acid β-oxidation protein Acox1 [39,41,57] are downregulated in *Mkp-1*^+/+^ mice after *E. coli* infection (Figure 10). It is worth noting that a recent study has shown that p38 inhibition enhanced parenteral nutrition-induced hepatic steatosis and attenuated the expression of Cpt1a, Acox1, and Ppargc1a in a rat model [58]. The dramatic decrease in p38 activity in *E. coli*-infected *Mkp-1*^+/+^ livers is consistent with the decreased expression of the Cpt1a, Acox1, and Ppargc1a as well as the increased triglyceride content in *E. coli*-infected *Mkp-1*^+/+^ mice.

Several factors can explain why *Mkp-1*^−/−^ mice failed to develop hyperlipidemia after *E. coli* infection like the *Mkp-1*^+/+^ mice did. First, it has been shown that *Mkp-1*^−/−^ mice on chow diet have lower adipose mass and higher metabolic activity with enhanced glucogenic activity under normal conditions [34]. Because *Mkp-1*^−/−^ mice had lower fat mass, *E. coli* infection might not be able to trigger the release of fatty acids and glycerol from adipose tissue into the circulation. It is also possible that exacerbated production of large quantities of pro- and anti-inflammatory cytokines in *Mkp-1*^−/−^ mice disrupted the normal triglyceride mobilization program in the adipose tissues. Alternatively, in the absence of Mkp-1, the livers might not be able to esterify the fatty acids released by adipose tissues into VLDL triglycerides in the liver. However, considering the normal Dgat2 protein levels in the livers of *E. coli*-infected *Mkp-1*^−/−^ mice (Figure 8), we think this is unlikely.

Previously, it has been shown that liver-specific *Mkp-1* deletion enhances gluconeogenesis [34,46,59,60]. Furthermore, it has been found that *Mkp-1*^−/−^ livers exhibit decreased Pparg and Srebf1 expression and increased Ppargc1a and Pck1 expression [46,60]. Our analyses, for the most part, corroborated their findings (Figure 6), although in our hands the difference in the liver expression of Ppargc1a and Srebf1 expression between *Mkp-1*^+/+^ and *Mkp-1*^−/−^ mice was not significant. Consistent with the elevated Pck1 expression in liver-specific *Mkp-1* knockout mice [46], we also found that liver Pck1 protein expression was significantly higher in the un-infected *Mkp-1*^−/−^ mice than in un-infected *Mkp-1*^+/+^ mice (Figure 11). The dramatically enhanced Pck1 expression suggests that in the absence of Mkp-1 animals adapt a more active ‘wasting’ program to meet their metabolic needs, partially explaining how *Mkp-1*^−/−^ mice utilize less glycogen following *E. coli* infection [10].

While our analyses shed insight into the mechanisms by which Mkp-1 facilitates hyperlipidemia during sepsis through a transcriptome lens, there are some limitations that need to be considered. RNA-seq performed here only gives a snap-shot of the transcriptome 24 h post-infection and prior to infection, the transitional transcriptome might be equally important in understanding the role of Mkp-1 in lipid metabolism during sepsis. Additionally, there were some disparities between RNA-seq data set, the qRT-PCR data, and/or Western blotting results. Despite these limitations, our analyses provide novel information on the function of Mkp-1 in the orchestration of lipid metabolic changes to facilitate immune defense. Our results clearly indicate that Mkp-1 plays an important role in the regulation of both the inflammatory response and metabolic programming during host defense against bacterial infection.

## 4. Materials and Methods

### 4.1. Experimental Animals and E. coli Infection

The present study was approved by the Institutional Animal Care and Use Committee of the Research Institute at Nationwide Children’s Hospital (01505AR, 9 January 2017). *Mkp-1*^−/−^ mice and the *Mkp-1*^+/+^ controls on a C57/129 mixed background [61] were kindly provided by Bristol-Myers Squibb Pharmaceutical Research Institute. In the absence of challenge, *Mkp-1*^−/−^ mice exhibit no sign of abnormality or growth retardation. All mice were housed with a 12 h alternating light-dark cycle at room temperature, and had free access to standard chow diet and water throughout the study. *Mkp-1*^−/−^ and *Mkp-1*^+/+^ mice were infected with or without *E. coli* as previously described [10]. Briefly, *E. coli* (O55:B5, ATCC 12014), purchased from American Type Culture Collection (Manassas, VA, USA), was grown in Luria broth at 37 °C for 18 h. The bacteria were pelleted by centrifugation, washed three times with phosphate-buffered saline (PBS), and finally suspended in PBS. *E. coli* was injected to the tail veins of mice at 2.5 × 10^7^ CFU/g body weight. Infected mice were euthanized 24 h later by pentobarbital over dose. Blood was collected through cardiac puncture, and coagulated blood was centrifuged to obtain serum. The liver of each mouse was excised with a small piece preserved in formalin for histological assessment and the rest of the liver snap-frozen in liquid nitrogen prior to storage at −80 °C.

### 4.2. Biochemical Assessment of Liver Lipid

Hepatic total lipid was extracted and determined gravimetrically as described [62]. Extracted liver lipid was solubilized to determine triglyceride and cholesterol spectrophotometrically using a triglyceride or cholesterol measuring kit (Pointe Scientific, Canton, MI, USA), according to the manufacturer’s instructions.

### 4.3. Histological Assessment of Liver Lipid Content

Hematoxylin and eosin (H&E) staining was conducted on paraffin-embedded liver sections (5 μm). The slides were evaluated blindly by a veterinary pathologist for histologically abnormalities. Images of ten randomly selected fields were captured (400× magnification) to assess liver lipid content using the established Brunt scoring system for assessing liver steatosis [63]. In brief, the liver lipid levels were scored as: grade 0 for <5% hepatocytes without lipid droplets; grade 1 for 5–33% of hepatocytes containing visible lipid droplets; grade 2 for fatty hepatocytes occupying 33–66% of the hepatic parenchyma; or grade 3 for >66% hepatocytes containing lipid droplets.

### 4.4. Liver RNA Extraction

Total RNA was extracted from frozen liver tissues using Trizol reagent (Thermo Fisher Scientific, Waltham, MA, USA), solubilized in UltraPure RNase/DNase-free water (Thermo Fisher Scientific), and quantified by using NanoDrop ND-1000 spectrophotometer (Marshall Scientific, Hampton, NH, USA).

### 4.5. RNA-Seq

For RNA-seq analyses, 1 µg total RNA was used as starting material. First, cytoplasmic and mitochondrial ribosomal RNAs were depleted using a NEBNext rRNA Depletion Kit (New England Biolabs, Ipswich, MA, USA), according to the manufacture’s recommendations. The samples were then digested with DNase I to remove contaminated genomic DNA, and purified using NEBNext RNA Sample Purification Beads (New England Biolabs). The RNA library was prepared using a NEBNext Ultra Directional RNA Library Prep Kit for Illumina (New England Biolabs). Briefly, RNA was fragmented, and then cDNAs were synthesized using random primers. The resulting double-strand cDNA was then subject to end-repair, adapter ligation, and PCR amplification to generate the library. The indexed RNA-seq libraries were quantitated by quantitative PCR, pooled with equimolar amounts and sequenced on an illumina HiSeq 3000 sequencer using a 2 × 125 cycle run, as previously described [64,65].

Following computational de-multiplexing, single end reads (50 bp) in the FASTQ format were generated. Quality control and adapter trimming were accomplished using the FastQC (version 0.11.3) and Trim Galore (version 0.4.0) software packages. Trimmed reads were mapped to the Genome Reference Consortium GRCm38 (mm10) murine genome assembly using TopHat2 (version 2.1.0), and feature counts were generated using HTSeq (version 0.6.1). Statistical analysis for differential expression was performed using the DESeq2 package (version 1.16.1) in R, with the default Benjamini-Hochberg *p* value adjustment method. Statistically significant differential expression thresholds included an adjusted *p* value <0.05 and an absolute value linear fold change of 2 or greater. Overrepresentation analysis for select gene sets comprising five or more members was determined using hypergeometric statistical testing (hyper function in R Documentation). Additionally, significantly impacted pathways were analyzed using Advaita Bio’s iPathwayGuide (https://www.advaitabio.com/ipathwayguide).

### 4.6. qRT-PCR

To confirm the result of RNA-seq, qRT-PCR was performed as previously described [62] with minor modifications. Briefly, genomic DNA was removed by digesting the total RNA with RQ1 RNase-Free DNase (Promega, Madison, WI, USA). Liver RNA was then reverse transcribed on PTC-200 DNA Engine Cycler (Bio-Rad, Hercule, CA, USA) with High-Capacity cDNA Reverse Transcription Kit (Applied Biosystems, Foster City, CA, USA). qRT-PCR was performed using PowerUp SYBR Green PCR Master Mix (Applied Biosystems) on a Realplex^2^ Mastercycler (Eppendorf, Hauppauge, NY, USA). All primers were synthesized by Integrated DNA Technologies (Coralville, IA, USA). Table 1 listed the primer sequences for the mRNAs of the following proteins, including proteins involved in fatty acid uptake, synthesis, oxidation, or their regulation: Cd36, Cpt1a/2, Acox1, Pck1, and Mtor. Hepatic mRNA expression of the genes of interest was calculated relative to 18s using the 2^−ΔΔ*C*t^ method [66].

### 4.7. Western Blotting

Frozen liver tissues were homogenized in lysis buffer (20 mM HEPES, pH7.4, 50 mM β-glycerol phosphate, 2 mM EGTA, 1 mM DTT, 10 mM NaF, 1 mM sodium orthovanadate, 10% glycerol, 1 mM PMSF, 2 µM leupeptin, 1.5 µM pepstatin, 0.3 µM aprotinin, and 50 nM microcystin-LR), using a Bullet Blender (Next Advance, Troy, NY, USA). Triton X-100 was then added to the homogenates to a final concentration of 1%, and the homogenates were incubated at 4 °C on a rotator at 300 rpm for 30 min. The homogenates were then centrifuged at 14,000× *g* for 10 min to collect the supernatants, and the protein concentrations were measured using a Protein Assay Kit (Bio-Rad). Extracted liver proteins were then separated on a NuPage 10% Bis-Tris gel (Invitrogen, Carlsbad, CA, USA), and transferred to nitrocellulose membranes. Membranes were probed for 1 h at room temperature or overnight at 4 °C with a primary antibody against a protein of interest. After washing three times with Tris-buffered saline with 0.1% Tween-20, membranes were incubated with a horseradish peroxidase-conjugated anti-rabbit or anti-mouse secondary antibody (GE Healthcare, Piscataway, NJ, USA). Mouse monoclonal antibodies against Cpt1a, Dgat2, Fasn, Pck1, and Scd1 as well as the rabbit polyclonal antibody against total p38 were purchased from Santa Cruz Biotechnology (Santa Cruz, CA, USA). Mouse monoclonal antibody against β-actin was purchased from Sigma-Aldrich (St. Louis, MO, USA). The rabbit polyclonal antibody against phospho-p38 was purchased from Cell Signaling (Danvers, MA, USA). Immunoreactive bands were developed using enhanced chemiluminescence reagent (Millipore, Burlington, MA, USA). The Western blot images were acquired using Epson Perfection 4990 PHOTO scanner (Epson, Long Beach, CA, USA) and intensities of the immunoreactive bands were measured by densitometry using the UVP Vison Works LS software (Upland, CA, USA).

### 4.8. Statistical Analysis

Data were analyzed using GraphPad Prism 7 (GraphPad Software; La Jolla, CA, USA). The main effects and their interaction were evaluated by two-way ANOVA with Tukey post-hoc test to detect group differences. In addition, two-tail student’s *t*-test was conducted to detect statistical difference of the biomarkers measured in only two groups. Variables with unequal variance were log-transformed to achieve a normal distribution. Differences with *p* < 0.05 were considered significant.

### 4.9. Data Availability

The main data supporting the findings of this study are available from the NCBI GENE Expression Omnibus GES122741. For additional information contact the corresponding author.

## Figures and Tables

**Table 1:** Primers used for qRT-PCR reactions.

Gene	Forward Primer	Reverse Primer
18s	GTAACCCGTTGAACCCCATT	CCATCCAATCGGTAGTAGCG
Acox1	CAGGAAGAGCAAGGAAGTGG	CCTTTCTGGCTGATCCCATA
Cd36	ATGGGCTGTGATCGGAACTG	TTTGCCACGTCATCTGGGTTT
Cpt1a	CAGAGGATGGACACTGTAAAGG	CGGCACTTCTTGATCAAGCC
Cpt2	GGATAAACAGAATAAGCACACCA	GAAGGAACAAAGCGGATGAG
Mtor	ATTCAATCCATAGCCCCGTC	TGCATCACTCGTTCATCCTG
Pck1	TCTCTGATCCAGACCTTCCAA	GAAGTCCAGACCGTTATGCAG
Pparg	CCAGAGTCTGCTGATCTGCG	GCCACCTCTTTGCTCTGATC

## Abbreviations

Acaa1	Acetyl-CoA acyltransferase 1
Acaca	Acetyl-CoA carboxylase α
Acacb	Acetyl-CoA carboxylase β
Acsl1	Long chain fatty acid-CoA ligase 1
Acot	Acyl-CoA thioesterase
Acox1	Acyl-CoA oxidase 1
ANOVA	Analysis of variance
Apo	Apolipoprotein
Cd36 CFU	Cluster of differentiation 36 Colony forming units
Cebp	CCAAT/enhancer-binding protein
Cpt	Carnitine palmitoyltransferase
Dgat2	Diglyceride acyltransferase 2
Fabp1	Fatty acid-binding protein 1
Fasn	Fatty acid synthase
Foxo1 JNK	Forkhead box protein O1 c-Jun N-terminal kinase
LPS	Lipopolysaccharide
Mkp-1	MAP kinase phosphatase-1
Mtor	Mammalian target of rapamycin
NF-κB	Nuclear factor-κB
PBS	Phosphate-buffered saline
Pck1/PEPCK-c	Phosphoenolpyruvate carboxykinase, cytosolic isoform
Plin	Perilipin
Pparg/PPARγ	Peroxisome proliferator-activated receptor γ
Ppargc1/PGC-1	PPARγ coactivator 1
Scd1	Stearoyl-CoA desaturase 1
qRT-PCR	Quantitative reverse transcription PCR
Srebf1/SREBP-1	Sterol regulatory element-binding transcription factor 1
TLR	Toll-like receptor
TNF-α	Tumor necrosis factor-α
VLDL	Very low-density lipoprotein
